# Global warming significantly increases the risk of Pierce’s disease epidemics in European vineyards

**DOI:** 10.1038/s41598-024-59947-y

**Published:** 2024-04-26

**Authors:** Àlex Giménez-Romero, Maialen Iturbide, Eduardo Moralejo, José M. Gutiérrez, Manuel A. Matías

**Affiliations:** 1 Instituto de Física Interdisciplinar y Sistemas Complejos (IFISC, CSIC-UIB), Campus UIB, 07122 Palma de Mallorca, Spain; 2 Instituto de Física de Cantabria (IFCA, CSIC-University of Cantabria), Avenida de los Castros, 39005 Santander, Spain; 3 Tragsa, Passatge Cala Figuera 6, 07009 Palma de Mallorca, Spain

**Keywords:** Ecological epidemiology, Climate-change impacts

## Abstract

Pierce’s disease (PD) is a vector-borne disease caused by the bacteria *Xylella fastidiosa*, which affects grapevines in the Americas. Currently, vineyards in continental Europe, the world’s largest producer of quality wine, have not yet been affected by PD. However, climate change may alter this situation. Here we incorporate the latest regional climate change projections into a climate-driven epidemiological model to assess the risk of PD epidemics in Europe for different levels of global warming. We found a significant increase in risk above $+\,2\,^{\circ }\hbox {C}$ in the main wine-producing regions of France, Italy and Portugal, in addition to a critical tipping point above $+\,3\,^{\circ }\hbox {C}$ for the possible spread of PD beyond the Mediterranean. The model identifies decreasing risk trends in Spain, as well as contrasting patterns across the continent with different velocities of risk change and epidemic growth rates. Although there is some uncertainty in model projections over time, spatial patterns of risk are consistent across different climate models. Our study provides a comprehensive analysis of the future of PD at multiple spatial scales (country, Protected Designation of Origin and vineyard), revealing where, why and when PD could become a new threat to the European wine industry.

## Introduction

Climate change is widely recognized as an important driver of shifts in the distribution and prevalence of plant diseases worldwide^1–6^. Although the impact of climate change on the distribution of plant diseases has been approached from various perspectives^7,8^, few studies have considered epidemiological dynamics in climate projections^9,10^. Modeling disease epidemics is a complex task, as they are emergent phenomena resulting from non-linear interactions between disease components. In addition, many of the processes involved in disease development also exhibit non-linear responses to changes in environmental variables^11,12^. This complexity is further exacerbated in the case of vector-borne plant diseases^13^. While climate primarily determines the potential geographic range of each organism in the pathosystem, the development of epidemic outbreaks depends on favorable host–pathogen–vector–climate interactions that drive transmission chains. Consequently, modeling the risk of vector-borne plant diseases implies delimiting their epidemiological niche rather than the ecological niche of their parts, as is commonly done.

The emergence of *X. fastidiosa* in Europe has led to a renewed interest in modeling vector-borne diseases, in particular Pierce's disease and the risk it poses to the European wine industry. Pierce’s disease (PD) is an endemic fatal disease of grapevines in the Americas, transmitted non-specifically by sap-feeding insect vectors belonging to sharpshooter leafhoppers (Hemiptera: Cicadellinae) and spittlebugs (Hemiptera: superfamily Cercopidae)^14^. In the US, PD causes huge economic losses to the wine sector estimated at 100 M$ per year in California alone^15^. The causative agent of PD is *Xylella fastidiosa* (Xf), a bacterium capable of colonizing the xylem vessels of more than 600 hosts, including important crops^16^. As a taxonomic unit, Xf comprises three recognized subspecies, *fastidiosa*, *multiplex*, and *pauca*, and more than 90 sequence types (i.e. genetic lineages) with distinct host ranges. Specifically, the Xf clonal lineage that causes Pierce’s disease (hereafter Xf$$_{\text{PD}}$$) also causes almond leaf scorch in California^17^.

Until the beginning of the twenty-first century, Xf was a pathogen officially restricted to the American continent^18^. In 2013, the involvement of Xf subsp. *pauca* in the massive death of ancient olive trees in Apulia, Italy, and its rapid spread raised alarm in European agriculture^19^. Today, all three Xf subspecies have been detected in the Balearic Islands (Spain), including Xf$_{\text{PD}}$, and several clonal lineages have been found in Corsica and the PACA region of France, Alicante (Spain), Tuscany (Italy) and Portugal^20–22^. Outside North America, Xf$_{\text{PD}}$ is only established on the islands of Mallorca and Taiwan, and has recently been detected in Israel, Lebanon and Portugal^23,24^. In all European outbreaks, the insect vector *Philaenus spumarius* is the main and almost unique carrier of Xf^25^.

Despite recent studies agree that the current risk of PD establishment in Europe is primarily confined to the Mediterranean basin^26–28^, its potential future progression is not yet clear. A larger set of available climate models is desirable to properly deal with the inherent uncertainty in the predictions. Some efforts have been made to characterize the geographical distribution of Xf-induced diseases in Europe under climate change, but these are limited to the use of species distribution models (SDMs) for the pathogen^29–31^ and the vector^27^, which have led to conflicting results. On the one hand, higher temperatures are expected to promote bacterial growth in susceptible crops in continental southern Europe, while on the other hand, these areas are progressively experiencing drier environmental conditions that are detrimental to vector populations^27^. Although the use of SDMs to predict the potential distribution of vector-borne plant diseases, provides good approximations, they are generally inadequate in the case of obligate pathogens such as Xf because they cannot separate the observed distribution of the pathogen from that of the vector. Furthermore, the potential distribution of the pathogen or the vector alone have no epidemiological significance for quantitative predictions of disease severity.

To overcome these limitations, here we employ a climate-driven epidemiological model that has previously been used to determine the risk of PD under current climatic conditions in wine-growing regions worldwide^28^. The model determines the spatio-temporal epidemic risk based on the spatial distribution of vectors, temperature-dependent bacterial growth and survival within hosts, and subsequent epidemiological dynamics by forcing the introduction of the pathogen and examining whether the disease can establish and spread from previous states under climatic conditions of the location. A very preliminary estimate of future PD risk was obtained by extrapolating the data using a linear regression. However, as mentioned in that paper^28^, this approach presents some evident flaws as it overlooks the role of nonlinearities in the model and does not take into account climate change scenarios. To assess the potential distribution and relative impact of PD on European vineyards under different levels of global warming, here we used state-of-the-art regional climate projections from the EURO-CORDEX initiative^32^. Our study takes into account uncertainties in climate projections and provides an updated and comprehensive assessment of PD risk in European wine-growing regions, addressing previous limitations. We posit that pest risk maps constructed from projections of epidemiological models driven by climate data provide a more realistic, quantitative and explanatory predictions than correlative and probabilistic models. Additionally, they offer valuable insights for anticipating and managing the potential impacts of PD and thus ensuring the resilience of viticulture despite future climate challenges.

## Methods

### Climate datasets

We used E-OBS version v21e^33^ as the reference observational climatic dataset, providing daily gridded data for Europe at a resolution of 0.1$^\circ$ ($\sim$ 10 km). Maximum and minimum temperature data was used to compute the MGDD and CDD indices involved in the growth and survival processes of the Xf$_{\text{PD}}$ pathogen (see “Climate-driven epidemiological model” section below). To calibrate the distribution models of *P. spumarius* capturing the widest possible range (North America and Europe), we used the ERA5-Land reanalysis^34^ due to its global (land) coverage and high resolution (0.1 $^\circ$, as E-OBS). Daily precipitation and daily minimum and maximum temperature data were retrieved to calculate the moisture index and the maximum temperature during spring index required for the vector suitability model (see “Vector climatic suitability” section below). Historical and future projections of both indexes were calculated using regional climate simulations from the state-of-the-art large high-resolution (0.11 $^\circ$) ensemble provided by EURO-CORDEX^35^. This dataset includes daily simulations of precipitation and temperatures from a large ensemble of Regional Climate Models (RCMs) driven by Global Climate Models (GCMs) from the CMIP5 project^36^. For this, we considered the RCP8.5 simulations for 40 combinations of GCMs-RCMs (Table 1). In order to calculate 20-year mean climatic indexes across the different global warming levels ($+\,1.5\,^{\circ }\hbox {C}$, $+\,2\,^{\circ }\hbox {C}$, $+\,3\,^{\circ }\hbox {C}$ and $+\,4\,^{\circ }\hbox {C}$), we relied on the time periods during which each CMIP5 driving model reaches the designated level within the RCP8.5 scenario (see^37^). This information is available at the IPCC WGI Atlas GitHub repository^38^.

**Table 1:** EURO-CORDEX GCM-RCM combinations used in this study.

	CNRM-CM5	EC-EARTH	HadGEM2-ES	IPSL-CM5A-MR	MPI-ESM-LR	NorESM1-M
CLMcom-CCLM4-8-17_v1	1	1	1		1	
DMI-HIRHAM5_v2	1	1	1			
GERICS-REMO2015_v2	1					
IPSL-WRF381P_v2	1					
KNMI-RACMO22E_v2	1		1			
SMHI-RCA4_v1	1	2	1	1		1
CLMcom-ETH-COSMO-crCLIM-v1-1_v1		2	1		1	1
DMI-HIRHAM5_v1		1		1	1	
IPSL-WRF381P_v1		1	1	1		1
KNMI-RACMO22E_v1		2		1	1	1
MOHC-HadREM3-GA7-05_v1		1	1		1	1
GERICS-REMO2015_v1				1		1
MPI-CSC-REMO2009_v1					1	
SMHI-RCA4_v1a					1	
DMI-HIRHAM5_v3						1

Numbers indicate the number of runs in each combination.

### Climate-driven epidemiological model

We used the model developed in Ref.^28^, which describes the initial exponential rise (or decrease) of infected plants at the onset of an epidemic based on two main features: the spatial distribution of the vector and the bacterial growth and survival processes mediated by temperature.Temperature-dependent PD symptom development were based on parametrization of the results obtained after inoculation 36 grapevine varieties^28^. In short, the density of vectors at a given site influences the number of new plants that will be inoculated with the bacterium, while the local temperature mediates the growth and survival processes of the in-plant bacterial population, leading to the initial inoculation to an infection or not. These temperature-driven growth and survival processes are described with the *accumulation* of two metrics denoted *Modified Growing Degree Days* (MGDD) and *Cold Degree Days* (CDD). The base function to compute the MGDD is proportional to the Xf temperature-dependent growth rate and is defined by,

$$\begin{aligned}&f(T)=\left\{ \begin{array}{lll} 0 &{} \text {if} &{} T<T_{\text {base}}\\ m_1\cdot T-b_1 &{} \text {if} &{} T_{\text {base}} \le T< T_1 \\ m_2\cdot T + b_2 &{} \text {if} &{} T_{1} \le T< T_{\text {opt}}\\ m_3\cdot T + b_3 &{} \text {if} &{} T_{\text {opt}} \le T < T_2 \\ m_4\cdot T + b_4 &{} \text {if} &{} T_2 \le T_{\text {max}} \\ 0 &{} \text {if} &{} T\ge T_{\text {max}} \end{array}\right. , \end{aligned}$$

where $T_{\text {base}}=12\,^{\circ }\hbox {C}$, $T_1=18$, $T_{\text {opt}}=28\,^{\circ }\hbox {C}$, $T_2=32$ and $T_{\text {max}}=35\,^{\circ }\hbox {C}$; the slopes are $m_1= 0.66$, $m_2=1$, $m_3=-1.25$ and $m_4=-\,3$ and the intercepts are $b_1=-\,8$, $b_2=-\,14$, $b_3=4$ and $b_4=105$. MGDD are then computed between $1{\text{st}}$ April and $31{\text{st}}$ October as

1 $$\begin{aligned} MGDD(t) = \frac{1}{24}\sum _{\tau \in t} f(T(\tau )), \end{aligned}$$

where $\tau$ is expressed in hours, *t* in years and we divide by 24 to obtain *MGDD*(*t*) in degree days. CDDs are computed between $1{\text{st}}$ November and $31^{\text{st}}$ March as

2 $$\begin{aligned} CDD(t)= \frac{1}{24}\sum _{\tau \in t} (6-T(\tau )) \ \quad \text {for} \quad T_i\le 6\,^{\circ }\text {C}. \end{aligned}$$

Altogether, the number of infected hosts is described by the following recurrence relation

3 $$\begin{aligned} I(t)=I(t-1)e^{\gamma (R_0-1)}\mathscr {F}(MGDD(t))\mathscr {G}(CDD(t)), \end{aligned}$$

where $\gamma$ is the death rate of infected vines, $R_0$ is the basic reproduction number of the disease and $\mathscr {F}(\cdot )$ and $\mathscr {G}(\cdot )$ are sigmoidal-like functions that relate the MGDD and CDD metrics to the probability of developing an infection from a given inoculation. Following^28^, $R_0$ in each cell *j* is related to the climatic suitability of the vector such that

4 $$\begin{aligned} R_0^j=R_0^*\cdot s_j=5\cdot s_j, \end{aligned}$$

$\gamma =0.2$ and the specific form of $\mathscr {F}(\cdot )$ and $\mathscr {G}(\cdot )$ is given by

5 $$\begin{aligned} \mathscr {F}(x)&= \frac{1}{1+e^{-0.012(x-975)}}, \end{aligned}$$

6 $$\begin{aligned} \mathscr {G}(x)&= \frac{2\cdot 10^7}{2\cdot 10^7 + x^3}. \end{aligned}$$

Finally, the risk index is derived as the effective growth rate of the infected population over the simulated time^28^,

7 $$\begin{aligned} r_j=\max \left\{ \frac{\ln (I_j(T) / I(0))}{\gamma (R_0^j-1)\cdot T}. -1\right\} . \end{aligned}$$

Because the typical time scale of the disease is 5 years ($1/\gamma$), we simulate periods of 7 years. If more years are available to simulate, we perform a re-introduction of the disease as a single infected plant in each cell after each 7-year period^28^.

### Model adaptation to daily temperature data

MGDD and CDD metrics were defined using hourly temperature data^28^. However, the E-OBS and CORDEX datasets only provide daily granularity. To overcome this limitation, we use a basic sinusoidal extrapolation relating maximum and minimum daily temperature to hourly temperatures,

8 $$\begin{aligned} T_h=\frac{T_{max}+T_{min}}{2} + \frac{T_{max}-T_{min}}{2}\sin (w\cdot h), \end{aligned}$$

with $w=2\pi /24$ and *h* ranging from 0 to 23. This approximation was validated with data from the national meteorological agency in Spain (AEMET). Basically, we used hourly tempererature data obtained from 50 meteorological stations in the period 2010–2020 and computed both MGDD and CDD using the full hourly data and only the daily maximum and minimum temperatures, in the latter case using Eq. (8). The results showed no differences between hourly or daily temperatures computation to estimate MGDD and CDD (Figs. S5, S6). Because the temporal resolution of the E-OBS and ERA-5 land data sets are different and are acquired using different methodologies, we evaluated the possible divergence between the MGDD and CDD estimates^28^. These metrics calculated with both data agreed, showing a mean difference of 54 and 17 units for MGDD and CDD, respectively, and a standard deviation of 200 units for both metrics (Fig. S7).

### Vector climatic suitability

**Figure 1:**
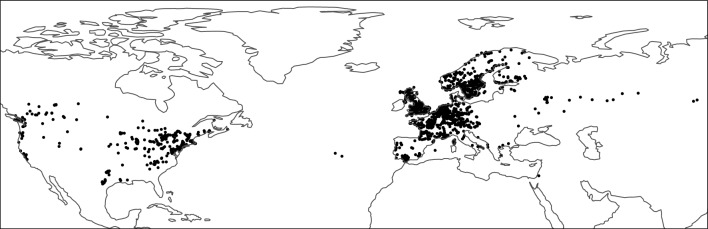
Training presence records for modeling the distribution of *Philaenus spumarius.*

Following^27^, we used the MaxEnt^39^ algorithm to calibrate the relationship of *P. spumarius* global occurrence (predictand) with the moisture index and the maximum temperatures during summer index (predictors) estimated from 2003 to 2022. Data of the presence records of *P. spumarius* were obtained from The Global Biodiversity Information Facility (GBIF)^40,41^ and different Spanish plant protection agencies and research institutions (“Instituto de Ciencias Agrarias” at CSIC, Madrid, Spain; “Servicio de Sanidad Vegetal de la Junta de Andalucía” based in Sevilla and Jaén, Andalucía, Spain; Sanidad Agrícola Econex S.L. based in Murcia, Spain), as reported in Ref.^27^. A total of 1652 presence records were used (Fig. 1), ensuring that there were no duplicated records within each cell of the climate layer grid.

In addition, we randomly generated pseudo-absences, also known as background points, using “The Three-Step” method proposed in Ref.^42^. This method incorporates a model performance criterion to determine the optimal sampling background extent, thereby ensuring that the model fitting was not adversely affected by the pseudo-absence sampling. Nevertheless, we accounted for the potential variability introduced by randomly selecting points from the background by performing 10 realizations of this sampling process. A total of 4956 pseudo-absences (three times the number of presences) were used in each realization.

**Figure 2:**
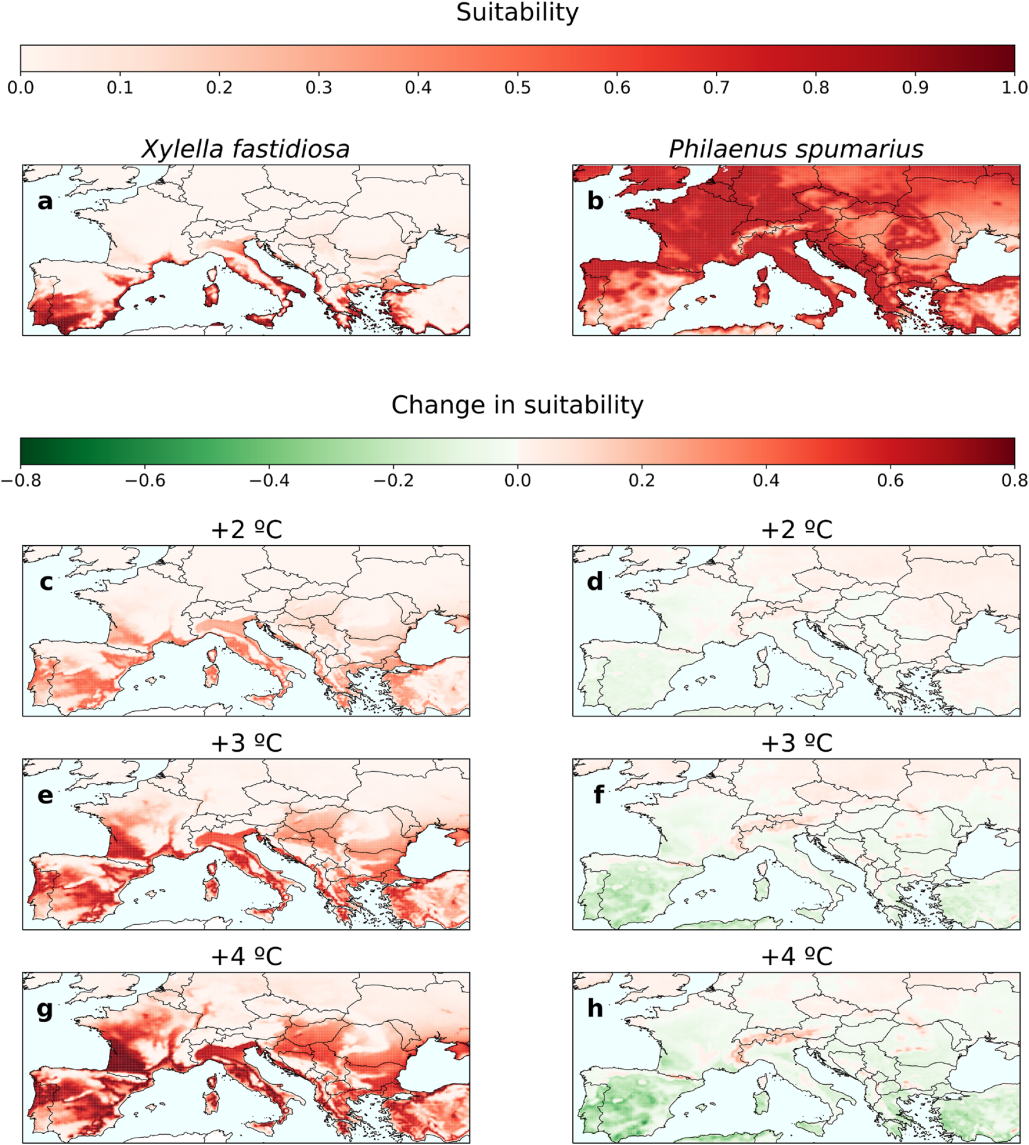
Changes in Xf$_{\text{PD}}$ and *P. spumarius* climatic suitability (i.e. probability of occurrence) under different climate projections compared to the current scenario (2003–2022). Current climatic suitability for the pathogen (**a**) and the vector (**b**). In an increasing temperature scenario ($+\,2\,^{\circ }\hbox {C}$ , $+\,3\,^{\circ }\hbox {C}$ and $+\,4\,^{\circ }\hbox {C}$) the climatic suitability for the pathogen geographically expands in southern Europe and moves northwards (**c,e,g**) while the climatic suitability for the vector decreases (**d,f,h**). The suitability values for each scenario correspond to a 20-year average.

Model evaluation was performed using a *k*-fold cross-validation approach (where $k = 10$) and the resulting AUCs (Area Under the ROC Curve) consistently exceeded 0.9 within the range of 0 to 1, with a value of 1 indicating perfect prediction and 0.5 indicating no discriminatory power (i.e. random guessing). Finally, the calibrated models were used to predict the suitability of *P. spumarius* in the reference historical period (2003–2022) and under increasing global warming scenarios (panels b, d, f, h in Fig. 2).

### Risk velocity

To assess the dynamic nature of the risk index and its spatial propagation, we introduced the concept of risk velocity, a metric analogous to the recently proposed concept of climatic velocity^43^. The risk velocity represents the rate at which the risk index changes over time and spreads across different locations. From an epidemiological perspective, risk velocity can be thought as the speed and direction the host would need to move to maintain its current risk conditions under climate change. Risk velocities were defined following the definition of climate velocity, as the ratio of the risk temporal trend and the risk spatial gradient in each cell. Thus, the units for the risk velocity correspond to kilometres per year (km/year). Risk velocities were computed using the VoCC R package^44,45^.

### Maps

**Figure 3:**
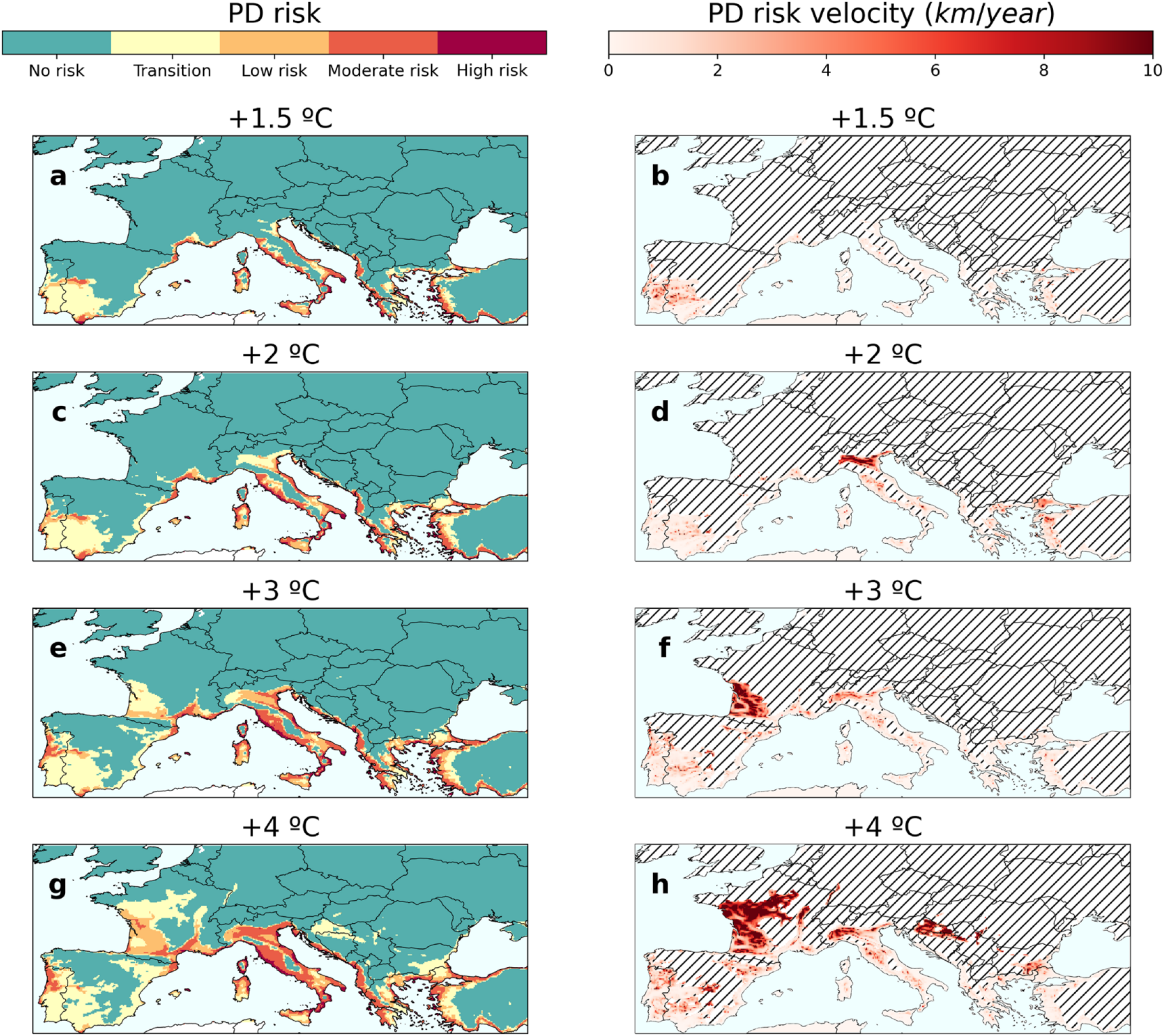
PD risk maps and associated risk velocities under different climate projections. (**a,b**) $+\,1.5\,^{\circ }\hbox {C}$ climate projection. (**c,d**) $+\,2\,^{\circ }\hbox {C}$ climate projection. (**e,f**) $+\,3\,^{\circ }\hbox {C}$ climate projection. (**g,h**) $+\,4\,^{\circ }\hbox {C}$ climate projection. Risk velocities have been calculated only in risk zones, $r > 0$, in each scenario. Hatched lines in panels (**b,d,f,h**) indicate no risk zones where risk velocities have not been calculated.

**Figure 4:**
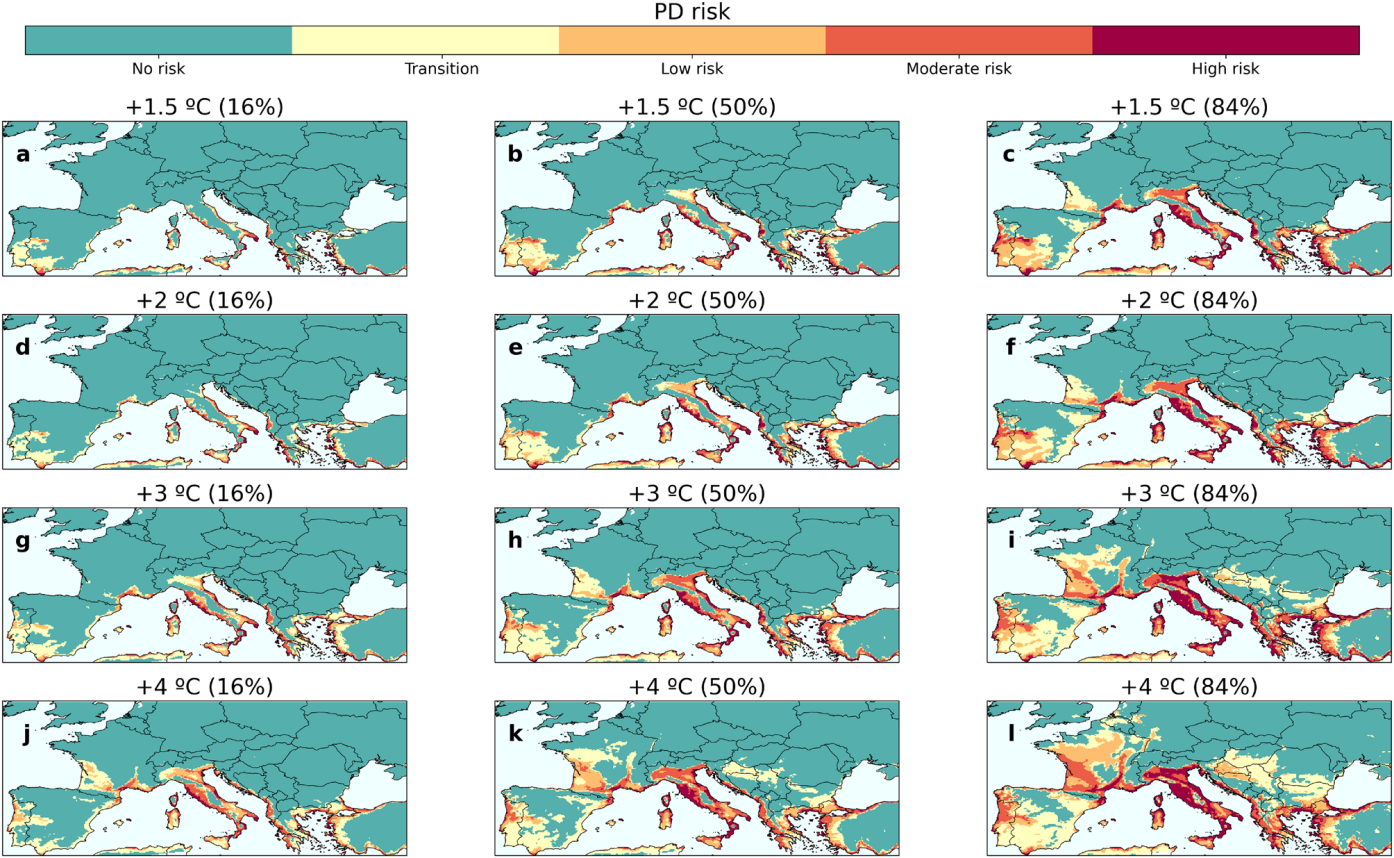
Uncertainty in PD risk projections for climate change warming levels. The maps show the result of the median risk values of the set of 40 regional climate model projections for each level of temperature increase (**b,e,h,k**) and the uncertainty of the projections considering the $1 \sigma$ deviations from the median, this is, the 16% (**a,d,g,j**) and 84% (**c,f,i,l**) percentiles. For each warming level (each row), the projected risk of PD is comprised between the results shown in the 16% percentile (first column) and those shown in the 84% percentile (last column) with 68% probability (1$\sigma$).

**Figure 5:**
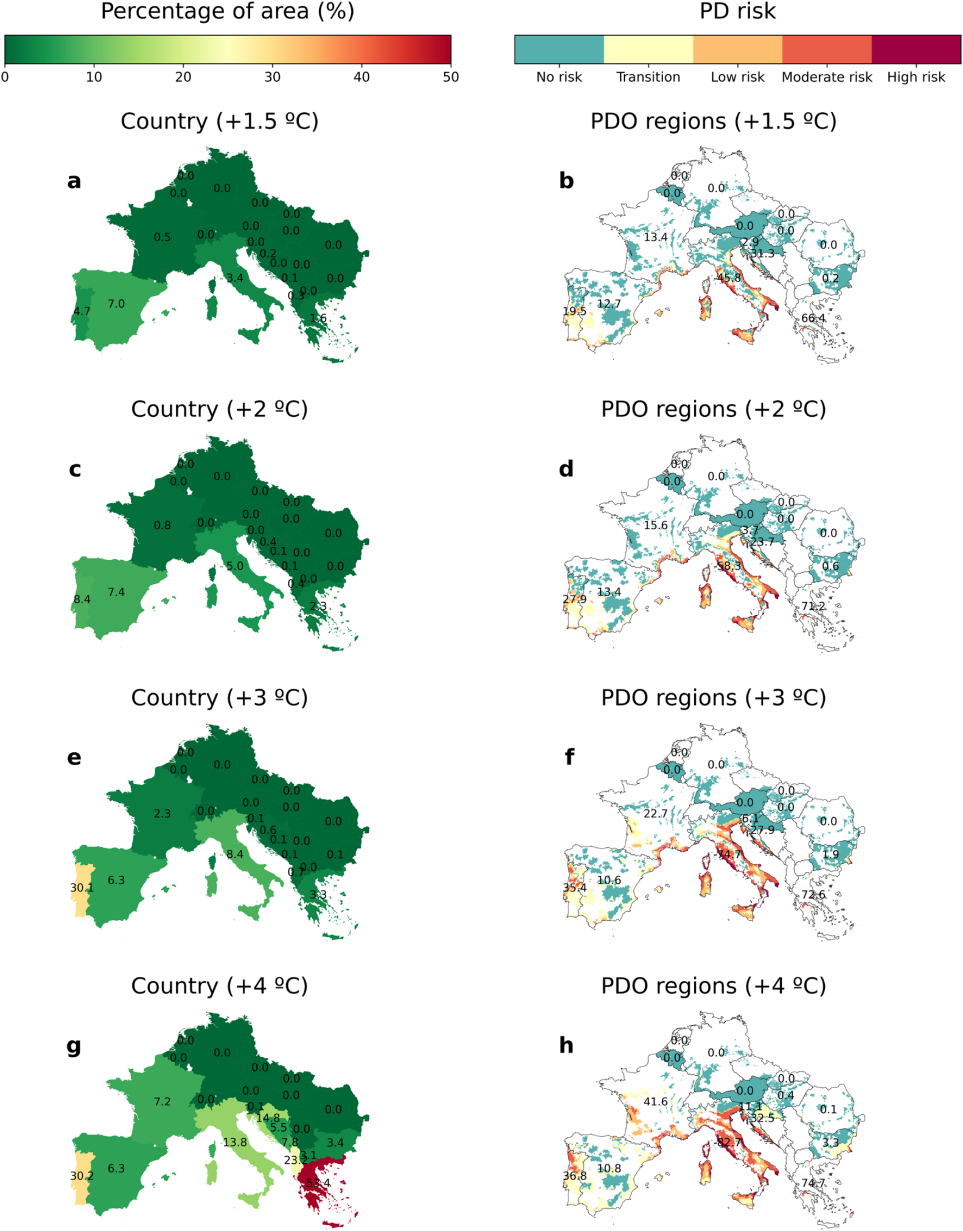
Multi-scale spatial analysis of PD future risk in Europe. (**a,c,e,g**) Percentage of country areas at risk ($r>0.1$) for each climate projection. (**b,d,f,h**) PD risk zones in Protected Designation of Origin (PDO) wine regions for each climate projection. PDO data was obtained from Ref.^51^. The corresponding interactive analysis at the vineyard level can be found in Ref.^50^.

The maps of all Figures in the paper (Figs. 1, 2, 3, 4, 5) and in the Supplementary Information file were made with Python 3.11^46^ using the package Cartopy v0.21.1^47,48^.

## Results

### Present and future climate suitability for *Xylella fastidiosa* (PD) and *Philaenus spumarius*

To gain a deeper understanding of how climate change affects each component of the pathosystem, we performed a separate analysis of climatic suitability conditions for Xf$_{\text{PD}}$ and *P. spumarius*. The thermal dependence of Xf$_{\text{PD}}$ growth and survival within the infected vine was mechanistically modeled by probability functions relating the accumulation of modified degree days (*MGDD*) and cold degree days (*CDD*) to symptom development and recovery, $\mathscr {F}(MGDD)$ and $\mathscr {G}(CDD)$, respectively (see “Methods” section). Climatic suitability for pathogen establishment was then determined by $\mathscr {F}(MGDD)\cdot \mathscr {G}(CDD)$, i.e. the overall probability of symptom development during the growing season and subsequent survival for overwintering infection (see “Methods” section). For *P. spumarius*, climatic suitability was modeled using an SDM based on a previous study^27^, with the climatic moisture index^49^ and spring maximum temperatures as key predictors (see Methods). Both analyses were evaluated under current (2003–2022) and future climate conditions considering scenarios of increasing global warming ($+\,1.5\,^{\circ }\hbox {C}$, $+\,2\,^{\circ }\hbox {C}$, $+\,3\,^{\circ }\hbox {C}$, and $+\,4\,^{\circ }\hbox {C}$) based on the latest generation of regional climate projections covering Europe^32^ (see “Methods” section).

Progressive global warming increases the accumulation of MGDD during the growing season and reduces the recovery rate (i.e. CDD) during winter, thus favouring the geographic expansion of the pathogen (Fig. 2, Supplementary Fig. S1). Conversely, increasing temperatures tend to reduce the climatic suitability of vectors in more arid areas of southern Europe leading to a progressive migration to higher areas and latitudes in continental regions in search of climatic refuge. These general trends hold for both organisms under the + 2, + 3 and $+\,4\,^{\circ }\hbox {C}$ temperature increase scenarios (Fig. 2).

The mechanistic approach to modelling pathogen establishment risk enables each of the two opposing directional processes of growth and survival (MGDD vs. CDD) to be appropriately weighted in the final result. For example, the Bordeaux region in western France has not been at risk due to low cumulative MGDD and low winter protection effect. In the transition from the $+\,1.5\,^{\circ }\hbox {C}$ scenario to the $+\,4\,^{\circ }\hbox {C}$ scenario, this area will experience a spectacular increase in risk mainly due to the expected summer warming (Fig. S2). Conversely, areas of Central Europe such as Hungary and Serbia already experience suitable conditions for pathogen growth in a $+\,1.5\,^{\circ }\hbox {C}$ scenario ($\mathscr {F}(MGDD) > 0.6$); however, cold winters tend to eliminate any potential summer infection $[\mathscr {G}(CDD) < 0.3]$ (Fig. S2). Climate change would further increase the growth of the pathogen and reduce the winter curing effect in Central Europe, ultimately exposing the region to Xf$_{\text{PD}}$ (Fig. S2).

### Pierce’s disease risk projections under climate change

The limited intersection between the climatic suitability ranges for the pathogen and the vector (Fig. 2a,b) suggests a marginal risk of PD epidemics in Europe. Since disease transmission requires a vector, the climate-suitability maps for *P. spumarius* indicate a lower risk and potential economic impact of *X. fastidiosa*-induced diseases on any host in southern Europe, particularly Spain, than previously predicted^30^. Realistic risk maps require a defined epidemiological framework to account for inter-annual climate variation and transmission in disease dynamics , in addition to accounting for changes in the distribution of climatic conditions favorable to the pathogen and vector (i.e. climatic suitability). Our epidemic risk model focuses on delimiting the disease dynamics by simulating an epidemic process in which the emergence of newly exposed hosts is influenced by the climatic suitability of the vector, while the transition to the infectious state is driven by the climatic suitability for Xf$_{\text{PD}}$ chronic infections. The effective growth rate of the infected host population over the simulated period is used to derive a risk index *r*, bounded between $-\,1$ and 1. Within this modeling framework, different risk categories naturally emerge: no risk ($r<-\,0.1$), transition zone ($-\,0.1\le r<0.1$), low risk ($0.1\le r<0.33$), moderate risk ($0.33\le r<0.66$) and high risk ($r\ge 0.66$). For further details see the “Methods” section and the original paper^28^ .

Global warming ($+\,1.5\,^{\circ }\hbox {C}$, $+\,2\,^{\circ }\hbox {C}$, $+\,3\,^{\circ }\hbox {C}$, and $+\,4\,^{\circ }\hbox {C}$) is expected to increase the risk of PD epidemics in southern Europe, with France, Italy and Portugal being particularly affected (Fig. 3, Supplementary Fig. S3). This general trend affects each of the risk categories under the different climate change scenarios (Fig. S4). Furthermore, we observed that a global temperature increase above $+3^{\circ }\hbox {C}$ represents a tipping point for the possible spread of PD beyond the Mediterranean (Fig. 3, Supplementary Fig. S3). To quantify the potential spread of PD, we calculated risk velocity, an index that allows us to identify areas where risk is changing or spreading rapidly (see “Methods” section). We found a consistent and notable increase in the mean risk velocity within most of the identified risk zones (Fig. 3, Supplementary Fig. S4), increasing from almost 1 km y$^{-1}$ to 5 km y$^{-1}$ as the temperature rises from a $+\,1.5\,^{\circ }\hbox {C}$ to a $+\,4\,^{\circ }\hbox {C}$ scenario (Table S1). This acceleration is evident when we compare that in the $+\,1.5\,^{\circ }\hbox {C}$ scenario, approximately 6% of the grid cells have risk velocities greater than 5 km y$^{\mathrm {-1}}$, while this value increases to 50% in the $+4^{\circ }\hbox {C}$ scenario (Table S1). Furthermore, our estimates of PD risk velocity are broadly consistent with estimates of the velocity of temperature change^43^, indicating that shifts in PD risk in our model adequately track climate change.

Figure 4 shows the uncertainty in the projections of the PD risk map, comparing the 16th and 84th percentiles ($1\sigma$) from the set of 40 regional climate models to the median risk map. The spatial distribution of PD risk is robust across models, while the uncertainty in the level of warming is bounded by a ± $1^{\circ }\hbox {C}$ increase (Fig. 4), e.g. the median spatial distribution of risk obtained for a $+\,3\,^{\circ }\hbox {C}$ warming level is expected to occur between the $+\,2\,^{\circ }\hbox {C}$ and the $+\,4\,^{\circ }\hbox {C}$ scenarios under a 1$\sigma$ confidence level. This means that, depending on the specific model, a given spatial distribution of risk (e.g., Fig. 4h) may manifest under a $+\,4\,^{\circ }\hbox {C}$ warming scenario in more conservative projections or a $+\,2\,^{\circ }\hbox {C}$ warming scenario in others, while most models project it for a $+\,3\,^{\circ }\hbox {C}$ warming level. This indicates a high degree of confidence in the location and projected severity of future outbreaks, but greater uncertainty in their timing.

In order to improve the accuracy of the risk maps and the relative impact of PD, we carried out a comprehensive analysis at multiple scales, from the national level to the regions with Protected Designation of Origin (PDO) and finally taking into account the distribution of vineyards. This approach allows us to disaggregate the results at different administrative levels to facilitate the design of risk management and the implementation of appropriate phytosanitary measures. Overall, our model simulations show a consistent increasing trend in the risk of PD in Europe under all climate change scenarios. The percentage of land area at risk in Europe increases from 0.32% in the $+\,1.5\,^{\circ }\hbox {C}$ scenario to 1.87% in the $+\,4\,^{\circ }\hbox {C}$, while the number of regions with PDO at risk increases from 18.17 to 47.32% . The vineyard area increases from 18.67 to 40.35% (Table S2). At country level Portugal and Greece face the highest overall risk, escalating from 12 and 2% of their area in the $+\,1.5\,^{\circ }\hbox {C}$ scenario to a striking 47% and 63% respectively in a $+\,4\,^{\circ }\hbox {C}$ scenario. In contrast, countries such as France and Italy experience a smaller but still significant increase in risk area, never exceeding the 20% threshold, while Spain, the third largest wine producer, shows a decreasing trend in risk areas above the $+\,2\,^{\circ }\hbox {C}$ scenario (Fig. 5, Table S3). Such contrasting patterns in PD risk between countries only emerge when using our modelling framework.

A different picture emerges when looking at the spatial distribution of PDO regions and vineyards. For example, PD risk within French and Italian PDO regions increases significantly from 13.4 and 45.8% in a $+\,1.5\,^{\circ }\hbox {C}$ scenario to 41.6% and 82.7% in a $+4^{\circ }\hbox {C}$ scenario (Fig. 5, Table S3), while the percentage of vineyard area at risk rises from 24.21 and 57.49% in a $+\,1.5\,^{\circ }\hbox {C}$ scenario to an astonishing 80% in a $+\,4\,^{\circ }\hbox {C}$ scenario. Important European PDOs would be at risk from a warming of $+\,2\,^{\circ }\hbox {C}$, such as parts of the southern Rhône Valley (Châteneuf du Pape), Provence and Languedoc in France, Penedés in Spain, Bairrada in Portugal, and Chianti and Brunello di Montalcino among others in Italy (see Supplementary Information). A detailed interactive analysis of the impact of PD in European PDO regions and vineyards is available on our website^50^.

## Discussion

Previous research has attempted to assess the potential geographic distribution of Xf$_{\text{PD}}$ subspecies, the insect vector *P. spumarius* and PD using species distribution models (SDMs) under future climates. While these climate-suitability-based predictions provide insights into the ecological niche of key disease players, bioclimatic correlative models neglect disease dynamics, a key factor in avoiding disease overestimation^28^. Unlike previous attempts, our approach integrates the compound effect of climate change in the pathosystem using a mechanistic epidemiological model to overcome these limitations. Unlike dimensionless climatic suitability indexes or disease probabilities used in SDMs, the risk index, *r*, in our model provides information on the expected growth rate in the event of an outbreak . Furthermore, the risk index is not fixed but varies annually depending on the weather conditions of the previous years. Inter-annual climate variability thus has an impact on disease dynamics, especially in areas where the risk index is lower. Another feature of our risk predictions is their lack of ambiguity, which should not be confused with certainty. Risk estimates are based on $R_0$, which depends on the insect vector population^52^, among other factors. Areas where $r<-\,0.1$ cannot sustain an outbreak, and the population of infected plants will decline over time. For example, our model clearly indicates that there is no risk of PD in the UK. This is not an arbitrary threshold; it is given by the epidemiological model. It is therefore very likely that the absence of PD in continental Europe is a consequence of low risk indices and that it has only become established in certain coastal areas since the late 1990s. On the contrary, the risk index in the Mediterranean islands has remained moderately high with little variation over the last 40 years^28^.

Because Pierce’s disease has only affected vineyards on the island of Mallorca^53^, little attention has been paid to the risk of it reaching continental vineyards. Our risk model indicates why this possibility was very low until the mid-90s, except for the Mediterranean islands^28^. In this work, we clearly show that with increasing temperatures PD will become a serious threat to important wine-growing areas in southern Europe that were not previously at risk. A key finding of our study is the identification of a tipping point for the risk of PD establishment at a global mean temperature increase of $+\,3\,^{\circ }\hbox {C}$. Beyond this threshold, the risk of PD spreading north of the Mediterranean region becomes remarkably higher, while the risk of PD epidemics in Portugal, Italy and France (Fig. 3) undergoes a significant quantitative leap. This suggests that as global temperatures continue to rise, the range of PD may expand into new territories. Indeed, the projected increase in risk velocities under higher warming scenarios further emphasizes the potential for rapid spread of PD into previously unaffected regions (Fig. 3).

Pest risk map projections are subject to uncertainties inherent in the variability of climate model predictions^54^. While previous studies on pathogen and vector distributions have been based on a limited number of climate models, our risk maps are based on the most modern set of regional climate projections produced by the EURO-CORDEX initiative, reflecting the state-of-the-art knowledge (Table 1). This allows us to adequately estimate the uncertainty of the resulting PD risk map projections for each temperature rise scenario. This confirms that although the spatial distribution of the risk of establishment is robust, there is an uncertainty of ± $1^{\circ }\hbox {C}$ in the level of warming (Fig. 4). The models are therefore fairly good at pointing where the increased risk will occur, but it is more difficult to know when it will be reached.

Overall, our results highlight the contrasting effect of climate change on PD risk distribution in Europe, revealing it as a multi-factor and multi-scale process (Table S3). Climate change has an opposite effect on each component of the pathosystem, enhancing areas of potential chronic PD infections while diminishing the suitable geographic range for the vector. At the same time, the characteristic spatial scale at which risk is assessed strongly influences conclusions. At the country level, there are significant variations in the extent of accumulated risk between different projections. However, when analyzed at a finer scale, such as at the level of PDO regions or vineyards, the results change completely. Countries that previously had marginal areas at risk now show a higher percentage of PDO regions and vineyard at risk. These results underlie the urgency of tailored mitigation and adaptation strategies to protect vineyards and PDOs, considering their specific spatial distribution and risk index, as well as the potential impacts of climate change.

Our results are influenced by the intrinsic uncertainty associated with the correlative models used to determine the spatial distribution of the vector, the epidemiological parameters, and the uncertainties in the climatic projections. Although the spatial resolution of our climate projections is considered to be high, it may not capture the complex micro-climate structure found in certain European wine-producing regions. Therefore, risk assessment results could differ locally with higher-resolution data. In addition, we have not considered the possible influence of climate change on latitudinal and altitudinal shifts in the distribution of European vineyards^55,56^, as this would only affect the calculation of the percentage of vineyard surface at risk but not the actual spatial distribution of risk. In any case, the risk estimates for the PDO regions include areas much larger than the areas of planted vines, which allows some margin in the adaptation and migration of the vineyards to different micro-climatic conditions. In addition, the PDO and vineyard databases used in this study are also have their own limitations. Future studies incorporating more refined modeling techniques, specific regional grapevine varieties, crop management and improved data resolution would enable a more nuanced understanding of PD risk and its potential impact at the local scale.

It is noteworthy that the mathematical framework employed in this study could be applied to other *Xylella fastidiosa* diseases, such as Almond Leaf Scorch Disease or Olive Quick Decline Syndrome and, more generally, to other vector-borne plant diseases. However, this requires the availability of some specific data and conducting some experiments. First, data for the temperature-dependent growth rate of the pathogen is needed to build the function that computes the MGDD. Then, symptom development experiments need to be carried out to build the $\mathscr {F}(MGDD)$ and $\mathscr {G}(CDD)$ functions that relate symptom development with temperature. Finally, the spatial distribution of the agent responsible for disease transmission is desired. Of course, using presence/absence data one can use SDM to obtain this spatial distribution.

Climate change is currently one of the biggest challenges for EU agricultural policy^57^. Quantitative regional predictions of climate change on emerging diseases, such as this one, provide a valuable and unambiguous tool for decision-making. In our approach to the problem, risk indexes not only include information on where or where not PD may become established, but also reflect the exponential growth rate of potential epidemics, which are directly related to their potential economic impact. In addition, risk indices and velocities provide a dynamic framework for assessing the feasibility of eradication efforts when Xf$_{\text{PD}}$ is detected in a new area, providing critical information for strategic crop protection. Our study evidences the need to selectively allocate more resources to surveillance and research on PD in southern European countries, considering the associated uncertainties. This strategic allocation of resources based on risk assessment can help to prioritise proactive measures and effectively manage the potential impact of PD in different European countries.

Our research highlights the complex dynamics of PD and its relationship with climate change. By adopting an interdisciplinary approach that integrates climate projections, epidemiological modelling, and spatial analysis, we provide valuable insights into the potential establishment and spread of PD in European wine-growing regions from the country to the vineyard levels. Our study demonstrates that an accurate assessment of the risk of PD establishment requires a nuanced understanding of the vector–plant–pathogen–climate system and the explicit consideration of the vineyard spatial setting. These findings can inform decision-making processes and support the development of effective strategies to mitigate the risks posed by PD and safeguard the future of viticulture in the face of a changing climate.

## Supplementary Information

Supplementary Information.

## Data Availability

PDO region georeference data was obtained from Ref.^51^. European vineyards georeferenced data was obtained from the Corine Land Cover^58^. MGDD, CDD and vector suitability data for the historical reference and the scenarios given by each warming level are available at Ref.^59^. We also provide the derived Xf$_{\text{PD}}$ suitability, PD risk and PD risk velocity data in the same repository. The developed webpage is freely accessible at Ref.^50^.

## Code availability

The code for the climate-driven epidemiological model is available in a GitHub repository^60^.
